# Supplementary material for: Cell-Projection Pumping of Fibroblast Contents into Osteosarcoma SAOS-2 Cells Correlates with Increased SAOS-2 Proliferation and Migration, as well as Altered Morphology
Source: Biomolecules. 2021 Dec 14;11(12):1875. doi: 10.3390/biom11121875 (PMC8699393; doi:10.3390/biom11121875)
Supplement: Supplementary file 1 [file biomolecules-11-01875-s001.zip › biomolecules-1483003(supplementary) new/Supplementary Materials/S1 Supplementary Materials.pdf]

Supplementary Materials For:
Cell-Projection Pumping of Fibroblast Contents into Osteosarcoma SAOS-2 Cells Correlates with Increased SAOS-2 Proliferation and Migration, as well as Altered Morphology

Swarna Mahadevan [1], James A Cornwell [1,2], Belal Chami [1,3], Elizabeth Kelly [1] and Hans Zoellner [1,4,5,6*]

[1] The Cellular and Molecular Pathology Research Unit, Oral Pathology and Oral Medicine, School of Dentistry, Faculty of Medicine and Health, The University of Sydney, Westmead Hospital, Westmead, NSW, 2145, Australia; smah9946@uni.sydney.edu.au (SM); elizabeth.kelly@sydney.edu.au (EK)
[2] Laboratory of Cancer Biology and Genetics, Center for Cancer Research, National Cancer Institute, Bethesda, MD, 20892, USA; cornwellja@mail.nih.gov
[3] Molecular Biomedicine, Charles Perkins Centre, The University of Sydney, NSW, 2006, Australia; belal.chami@sydney.edu.au
[4] Biomedical Engineering, Faculty of Engineering, The University of Sydney, NSW, 2006, Australia
[5] Graduate School of Biomedical Engineering, University of NSW, NSW, 2052, Australia
[6] Strongarch Pty Ltd, Sydney, NSW, 2000, Australia
* Correspondence: hans.zoellner@strongarch.com

S1. Supplementary Movie Legend and Tables

Supplementary Movie Legend. Movie SM1. Time-lapse recordings of SAOS-2 prelabelled with DiO (green fluorescence) and co-cultured for 88h with HDFs prelabelled with DiD (red fluorescence), showing phase contrast images at 15 minute intervals (left panel) and fluorescence images at 4h intervals (right panel). The visual field shown is one of nine separate contiguous fields, that comprised a 3 x 3 grid. SAOS-2 were recognized by their generally smaller size and green labelling, which in this experiment varied from being conspicuous in some cells to very modest in others. HDFs were generally much larger cells and had clear red fluorescence. Cell borders were generally more clear in phase contrast images, with fluorescence distribution within cells being punctate and often concentrated more towards the centres of cells. The visual impression was that HDFs migrated generally more quickly compared with SAOS-2. Mitosis was frequently seen, and this was accompanied by increased crowding of cells. Some SAOS-2 acquired appreciable red fluorescence from HDFs during co-culture. Casual examination of this and other time-lapse recordings, was insufficient to properly comprehend the numerous simultaneous events that occurred. Single-cell tracking permitted systematic numeric analysis of this complex system.

Table S1. The number of cells tracked in co-cultures of SAOS-2 with HDFs, according to cell type, generation (Gen.), experiment, and apoptosis, indicated by numbers in brackets (n).

	The Number of Co-Cultured SAOS-2 Tracked					
	Starting Gen.	1st Gen.	2nd Gen.	3rd Gen.	4th Gen.	Total
Experiment a	52	88 (1)	46 (1)			186 (2)
Experiment b	234 (19)	312 (16)	86			632 (35)
Experiment c	98 (10)	81 (13)	4			183 (23)
Experiment d	33 (4)	44 (2)	40 (1)	8		125 (7)
Experiment e	51	99 (6)	121 (4)	31 (1)		302 (11)
Experiment f	63 (6)	50 (8)	10			123 (14)
Experiment g	76 (14)	112 (6)	103 (2)	4		295 (22)
Total	607 (53)	786 (52)	410 (8)	43 (1)		1,846 (114)
	The Number of Co-Cultured HDFs Tracked					
	Starting Gen.	1st Gen.	2nd Gen.	3rd Gen.	4th Gen.	Total
Experiment a	37	30	16	6		89
Experiment b	168 (4)	120 (3)	26 (1)	4		318 (8)
Experiment c	105 (4)	28 (1)	10 (1)			143 (6)
Experiment d	31 (1)	33	32 (1)	4		100 (2)
Experiment e	70	66 (1)	42(1)	18	2	198 (2)
Experiment f	53					53
Experiment g	59	24	8			91
Total	523 (9)	301 (5)	134 (4)	32	2	992 (18)

Considering all co-cultured cells across all 7 experiments, a total of 2,838 co-cultured cells were tracked, amongst which 132 became apoptotic. The number of mitotic events is clear from the number of cells tracked in successive generations. Note that very occasionally, a daughter cell was quickly lost from vision and so not tracked, accounting for odd numbers in columns for generations 1 onwards, that contain otherwise paired sister cells.

Table S2. The number of control SAOS-2 and HDFs tracked, according to cell type, generation (Gen.), experiment, and apoptosis, indicated by numbers in brackets (n).

	The Number of Control SAOS-2 Tracked					
	Starting Gen.	1st Gen.	2nd Gen.	3rd Gen.	4th Gen.	Total
Experiment a	46 (6)	62	14			122 (6)
Experiment b	147 (10)	176 (9)	17			340 (19)
Experiment c	129 (8)	154 (15)	23 (1)			306 (24)
Experiment d	38 (3)	56 (3)	54 (6)	6		154 (12)
Experiment e	88 (4)	146 (10)	120 (4)	14	2	370 (18)
Experiment f	50 (8)	30 (2)				80 (10)
Experiment g	42	66 (9)	34			142 (9)
Total	540 (39)	690 (48)	262 (11)	20	2	1,514 (98)

	The Number of Control HDFs Tracked					
	Starting Gen.	1st Gen.	2nd Gen.	3rd Gen.	4th Gen.	Total
Experiment a	37	39	24	3	2	105
Experiment b	78 (2)	31	6			115 (2)
Experiment c	118 (2)	36				154 (2)
Experiment d	20	16	8	6		50
Experiment e	99	108 (2)	61 (1)	8	2	278 (3)
Experiment f	45 (3)					45 (3)
Experiment g	61 (3)	37	4			102 (3)
Total	458 (10)	267 (2)	103 (1)	17	4	849 (13)

Considering all co-cultured cells across all 7 experiments, a total of 2,363 control cells were tracked, amongst which 111 became apoptotic. The number of mitotic events is clear from the number of cells tracked in successive generations. Note that very occasionally, a daughter cell was quickly lost from vision and so not tracked, accounting for odd numbers in columns for generations 1 onwards, that contain otherwise paired sister cells.

Table S3. Median values for cell-profile area, cell circularity and cell migration velocity in control SAOS-2 and HDFs cultured in isolation. Statistical significance of differences between SAOS-2 and HDFs as per Mann Whitney U Test are shown for individual experiments, as well as for all experiments considered together as per Wilcoxon Signed Rank Test (*Sig.*).

		Median Cell-Profile Area (μm^2)		Median Cell Circularity		Median Cell Migration Velocity (μm/day)	
		SAOS-2	HDFs	SAOS-2	HDFs	SAOS-2	HDFs
Experiment a	Medians	1,271	4,179	0.79	0.35	94	456
	Sig.	*< 0.0001*		*< 0.0001*		*< 0.0001*	
Experiment b	Medians	1,677	11,978	0.63	0.18	84	287
	Sig.	*< 0.0001*		*< 0.0001*		*< 0.0001*	
Experiment c	Medians	1,524	6,695	0.81	0.34	58	244
	Sig.	*< 0.0001*		*< 0.0001*		*< 0.0001*	
Experiment d	Medians	1,359	4,333	0.67	0.36	86	327
	Sig.	*< 0.0001*		*< 0.0001*		*< 0.0001*	
Experiment e	Medians	1,380	3,785	0.85	0.38	81	401
	Sig.	*< 0.0001*		*< 0.0001*		*< 0.0001*	
Experiment f	Medians	1,084	2,923	0.87	0.31	67	230
	Sig.	*< 0.0001*		*< 0.0001*		*< 0.0001*	
Experiment g	Medians	1,070	4,094	0.71	0.37	92	316
	Sig.	*< 0.0001*		*< 0.0001*		*< 0.0001*	
Medians of Experiments	Medians	1,359	4,179	0.79	0.35	84	316
	Sig.	*0.0156*		*0.0156*		*0.0156*	

SAOS-2 had lower cell-profile area, higher circularity and lower cell migration velocity compared with HDFs, in all experiments.

Table S4 Kendall's tau of correlation between cell-profile area of tracked SAOS-2 and absolute fluorescence acquired from co-cultured HDFs (Fa), as well as with compensation for halving of fluorescence from mother cells by cell division (Fmc). Results for all experiments are shown, considering: all generations of cells together; starting and first generations together; and where a second generation of cells was present, starting, first and second generations together. Statistical significance is given, where *NS* indicates 'not significant' to p < 0.05. Where statistical significance was approached but not reached, the calculated p value is given (*NS (p value)*).

	**Kendall's tau of Correlation Between Cell-Profile Area and Receipt of HDF Fluorescence by SAOS-2**	
	Fa	Fmc
**Experiment a**		
Kendall's tau of all Generations Considered Together	0.55	0.53
Statistical Significance of the Above	*< 0.0001*	*< 0.0001*
Kendall's tau of Starting and First Generations Considered Together	0.54	0.47
Statistical Significance of the Above	*< 0.0001*	*< 0.0001*
**Experiment b**		
Kendall's tau of all Generations Considered Together	0.15	0.11
Statistical Significance of the Above	*< 0.0001*	*< 0.0001*
Kendall's tau of Starting and First Generations Considered Together	0.14	0.10
Statistical Significance of the Above	*< 0.0001*	*0.0003*
**Experiment c**		
Kendall's tau of all Generations Considered Together	0.76	0.68
Statistical Significance of the Above	*< 0.0001*	*< 0.0001*
Kendall's tau of Starting and First Generations Considered Together	0.76	0.67
Statistical Significance of the Above	*< 0.0001*	*< 0.0001*
**Experiment d**		
Kendall's tau of all Generations Considered Together	0.51	0.45
Statistical Significance of the Above	*< 0.0001*	*< 0.0001*
Kendall's tau of Starting and First Generations Considered Together	0.49	0.48
Statistical Significance of the Above	*< 0.0001*	*< 0.0001*
Kendall's tau of Starting, First and Second Generations Considered Together	0.49	0.44
Statistical Significance of the Above	*< 0.0001*	*< 0.0001*
**Experiment e**		
Kendall's tau of all Generations Considered Together	0.13	0.08
Statistical Significance of the Above	*0.001*	*0.04*
Kendall's tau of Starting and First Generations Considered Together	0.09	0.035
Statistical Significance of the Above	*NS (0.098)*	*NS*
Kendall's tau of Starting, First and Second Generations Considered Together	0.11	0.07
Statistical Significance of the Above	*0.0059*	*0.072*
**Experiment f**		
Kendall's tau of all Generations Considered Together	0.55	0.51
Statistical Significance of the Above	*< 0.0001*	*< 0.0001*
Kendall's tau of Starting and First Generations Considered Together	0.59	0.53
Statistical Significance of the Above	*< 0.0001*	*< 0.0001*
**Experiment g**		
Kendall's tau of all Generations Considered Together	0.54	0.41
Statistical Significance of the Above	*<0.0001*	*< 0.0001*
Kendall's tau of Starting and First Generations Considered Together	0.5	0.4
Statistical Significance of the Above	*< 0.0001*	*< 0.0001*
Kendall's tau of Starting, First and Second Generations Considered Together	0.54	0.41
Statistical Significance of the Above	*< 0.0001*	*< 0.0001*

There was strong correlation of cell-profile area with HDFs fluorescence. Correlation with Fa was stronger than for Fmc (p < 0.016, Wilcoxon Signed Rank Test). Inclusion of progressive generations of cells in calculations for tau had little effect on the strength or statistical significance of the correlation. Data indicate no heritability of the effect of fluorescence uptake past cell division, with an index of heritability of 0.

Table S5. Median values for cell-profile area in control SAOS-2 and HDFs cultured in isolation, as well as in co-cultures across all experiments. Statistical significance is shown as per Mann Whitney U Test of comparisons between co-cultured cells and control cells cultured in isolation. Where statistical significance was not reached, '*NS*' is shown for 'not significant'.

		Median Cell-Profile Area (μm^2)	
		SAOS-2	HDFs
Experiment a	Isolated Control Culture	1,271	4,179
	Co-Culture	1,677	3,850
	Statistical Significance	*0.0033*	*NS*
Experiment b	Isolated Control Culture	1,677	11,978
	Co-Culture	1,320	2,991
	Statistical Significance	*< 0.0001*	*< 0.0001*
Experiment c	Isolated Control Culture	1,524	6,695
	Co-Culture	1,124	4,172
	Statistical Significance	*< 0.0001*	*< 0.0001*
Experiment d	Isolated Control Culture	1,359	4,333
	Co-Culture	1,512	3,499
	Statistical Significance	*NS*	*0.0170*
Experiment e	Isolated Control Culture	1,380	3,785
	Co-Culture	1,064	3,146
	Statistical Significance	*< 0.0001*	*0.0069*
Experiment f	Isolated Control Culture	1,084	2,923
	Co-Culture	1,067	3,179
	Statistical Significance	*NS*	*NS*
Experiment g	Isolated Control Culture	1,070	4,094
	Co-Culture	1,366	5,032
	Statistical Significance	*0.0023*	*NS*
Medians of Experiments	Isolated Control Culture	1,359	4,179
	Co-Culture	1,320	3,499

SAOS-2 had lower cell-profile area compared with HDFs, irrespective if in co-culture or in isolated control cultures ($p < 0.0001$ within all individual experiments, Mann Whitney U Test; $p < 0.016$ across experiments Wilcoxon Signed Rank Test). There was no consistent difference between control and co-cultured SAOS-2 with regard to cell-profile area. In 5 of 7 experiments, co-cultured HDFs had lower cell-profile area compared with HDFs control cultures (experiments a, b, c, d, e), and this reached statistical significance in 4 of these ($p < 0.02$, Mann Whitney U Test), although considering all experiments together, this was not statistically significant as assessed by Wilcoxon Signed Rank Test.

Table S6. Kendall's tau for correlation in differences between paired sister cells for cell-profile area and absolute fluorescence (Fa). Statistical significance *(Stat. Sig.)* is indicated to p < 0.05, and *'NS'* for 'not significant' is recorded where statistical significance was not reached.

	First Generation Cells		Second Generation Cells		First and Second Generations of Cells Considered Together	
	Kendall's tau	*Stat. Sig.*	Kendall's tau	*Stat. Sig.*	Kendall's tau	*Stat. Sig.*
Experiment a	0.31	*0.0031*	0.59	*< 0.0001*	0.41	*0.0001*
Experiment b	0.15	*< 0.0001*	0.12	*NS*	0.15	*0.003*
Experiment c	0.63	*< 0.0001*				
Experiment d	0.39	*0.011*	0.49	*0.0018*	0.45	*< 0.0001*
Experiment e	0.54	*NS*	-0.087	*NS*	0.02	*NS*
Experiment f	0.71	*< 0.0001*	0.6	*NS*	0.68	*< 0.0001*
Experiment g	0.36	*< 0.0001*	0.5	*< 0.0001*	0.43	*< 0.0001*

Divergence from an expected correlation of 0 was statistically significant as per One Sample Wilcoxon Test, for first generation cells (p < 0.016) and when first and second generation cells were considered together (p < 0.032); and approached but did not reach statistical significance for second generation cells (p = 0.063). Strong correlation was seen across experiments with exception of experiment 'e', where there was very weak negative correlation for second generation cells, and the correlation for first generation cells although strong, was not statistically significant.

Table S7. Kendall's tau of correlation between cell circularity of tracked SAOS-2 and absolute fluorescence acquired from co-cultured HDFs (Fa), as well as with compensation for halving of fluorescence from mother cells by cell division (Fmc). Results for all experiments are shown, considering: all generations of cells together; starting and first generations together; and where a second generation of cells was present, starting, first and second generations together. Statistical significance is given, where *NS* indicates 'not significant' to $p < 0.05$. Where statistical significance was approached but not reached, the calculated p value is given (*NS (p value)*).

	**Kendall's tau of Correlation Between Cell Circularity and Receipt of HDF Fluorescence by SAOS-2**	
	Fa	Fmc
**Experiment a**		
Kendall's tau of all Generations Considered Together	-0.14	-0.18
Statistical Significance of the Above	*0.0058*	*0.0002*
Kendall's tau of Starting and First Generations Considered Together	-0.19	-0.22
Statistical Significance of the Above	*0.0009*	*< 0.0001*
**Experiment b**		
Kendall's tau of all Generations Considered Together	-0.03	-0.05
Statistical Significance of the Above	*NS*	*NS (0.051)*
Kendall's tau of Starting and First Generations Considered Together	-0.02	-0.05
Statistical Significance of the Above	*NS*	*NS (0.063)*
**Experiment c**		
Kendall's tau of all Generations Considered Together	-0.36	-0.37
Statistical Significance of the Above	*< 0.0001*	*<0.0001*
Kendall's tau of Starting and First Generations Considered Together	-0.35	-0.36
Statistical Significance of the Above	*< 0.0001*	*< 0.0001*
**Experiment d**		
Kendall's tau of all Generations Considered Together	-0.11	-0.12
Statistical Significance of the Above	*NS (0.06)*	*0.045*
Kendall's tau of Starting and First Generations Considered Together	-0.24	-0.23
Statistical Significance of the Above	*0.0024*	*0.0032*
Kendall's tau of Starting, First and Second Generations Considered Together	-0.15	-0.15
Statistical Significance of the Above	*0.02*	*0.015*
**Experiment e**		
Kendall's tau of all Generations Considered Together	-0.18	0.03
Statistical Significance of the Above	*NS (0.14)*	*NS*
Kendall's tau of Starting and First Generations Considered Together	0.10	0.16
Statistical Significance of the Above	*NS (0.06)*	*0.0026*
Kendall's tau of Starting, First and Second Generations Considered Together	-0.04	0.07
Statistical Significance of the Above	*NS*	*NS (0.096)*
**Experiment f**		
Kendall's tau of all Generations Considered Together	-0.08	-0.14
Statistical Significance of the Above	*NS*	*0.019*
Kendall's tau of Starting and First Generations Considered Together	-0.12	-0.16
Statistical Significance of the Above	*0.061*	*0.012*
**Experiment g**		
Kendall's tau of all Generations Considered Together	-0.19	-0.15
Statistical Significance of the Above	*< 0.0001*	*< 0.0001*
Kendall's tau of Starting and First Generations Considered Together	-0.22	-0.18
Statistical Significance of the Above	*< 0.0001*	*0.0003*
Kendall's tau of Starting, First and Second Generations Considered Together	-0.18	-0.15
Statistical Significance of the Above	*< 0.0001*	*< 0.0001*

There was weak inverse correlation of cell circularity with HDFs fluorescence in all but one experiment, where the reverse effect was seen (Exp. e). Correlations were generally strongest considering starting and first generations together, suggestive of confounding effects of cell crowding at later time points. For this reason, analysis focused on starting and first generation cells pooled. The strength of the inverse correlation across Fa and Fmc varied amongst experiments. Tau values Fmc were higher than Fa in 4 experiments (a, b, c and f), while the reverse was the case in 2 experiments (d and g), and experiment 'e' was equivocal. Although statistically compelling within a number of individual experiments, differences between experiments were such that no statistically significant result could be attributed to these general patterns. Overall, data indicate negative correlation of cell circularity with uptake of fibroblast fluorescence, with moderately strong persistence of circularity past mother cell division (index of persistence of 0.71).

Table S8. Median values for cell circularity in control SAOS-2 and HDFs cultured in isolation, as well as in co-cultures across all experiments. Statistical significance is shown as per Mann Whitney U Test of comparisons between co-cultured cells and control cells cultured in isolation. Where statistical significance was not reached, '*NS*' is shown for 'not significant'.

		Median Cell Circularity	
		SAOS-2	HDFs
Experiment a	Isolated Control Culture	0.79	0.35
	Co-Culture	0.81	0.38
	Statistical Significance	*NS*	*NS*
Experiment b	Isolated Control Culture	0.63	0.18
	Co-Culture	0.57	0.37
	Statistical Significance	*0.0247*	*< 0.0001*
Experiment c	Isolated Control Culture	0.81	0.34
	Co-Culture	0.65	0.37
	Statistical Significance	*< 0.0001*	*0.0303*
Experiment d	Isolated Control Culture	0.67	0.36
	Co-Culture	0.68	0.45
	Statistical Significance	*NS*	*0.0013*
Experiment e	Isolated Control Culture	0.85	0.38
	Co-Culture	0.73	0.41
	Statistical Significance	*< 0.0001*	*0.0069*
Experiment f	Isolated Control Culture	0.87	0.31
	Co-Culture	0.85	0.30
	Statistical Significance	*NS*	*NS*
Experiment g	Isolated Control Culture	0.71	0.37
	Co-Culture	0.67	0.33
	Statistical Significance	*0.0317*	*NS*
Medians of Experiments	Isolated Control Culture	0.79	0.35
	Co-Culture	0.68	0.37

SAOS-2 had higher cell-circularity compared with HDFs, irrespective if in co-culture or in isolated control cultures ($p < 0.0001$ within all individual experiments, Mann Whitney U Test; $p < 0.016$ across experiments Wilcoxon Signed Rank Test). SAOS-2 had lower cell circularity in co-culture compared with controls in 5 of 7 experiments (experiments b, c, e, f, g), reaching statistical significance in three experiments ($p < 0.025$, Mann Whitney U Test), but this was not significant when all experiments were considered together ($p > 0.05$, Wilcoxon Signed Rank Test). The reverse was seen in 5 experiments (a, b, c, d, e) comparing co-cultured with control HDFs, reaching statistical significance in 4 experiments ($p < 0.035$, Mann Whitney U Test), but once more, not when all experiments were considered together ($p > 0.05$, Wilcoxon Signed Rank Test).

Table S9. Kendall's tau for correlation of differences between paired sister cells for cell circularity and absolute fluorescence (Fa). Statistical significance *(Stat. Sig.)* is indicated to $p < 0.05$, and *'NS'* for 'not significant' is recorded where statistical significance was not reached. Where statistical significance was approached but not reached, calculated significance is given (*NS (p value)*).

	First Generation Cells		Second Generation Cells		First and Second Generations of Cells Considered Together	
	Kendall's tau	*Stat. Sig.*	Kendall's tau	*Stat. Sig.*	Kendall's tau	*Stat. Sig.*
Experiment a	0.06	*NS*	-0.08	*NS*	0.01	*NS*
Experiment b	-0.13	*NS*	-0.04	*NS*	-0.02	*NS*
Experiment c	0.04	*NS*				
Experiment d	-0.03	*NS*	0.18	*NS*	0.08	*NS*
Experiment e	0.01	*NS*	0.16	*NS*	0.12	*0.046*
Experiment f	0.60	*NS*	0.15	*NS*	0.15	*NS*
Experiment g	0.18	*NS (0.055)*	0.08	*NS*	0.14	*0.03*

Weak correlation was seen in only two experiments (e, g) when all cells were considered together.

Table S10. Kendall's tau of correlation between cell migration velocity of tracked SAOS-2 and absolute fluorescence acquired from co-cultured HDFs (Fa), as well as with compensation for halving of fluorescence from mother cells by cell division (Fmc). Results for all experiments are shown, considering: all generations of cells together; starting and first generations together; and where a second generation of cells was present, starting, first and second generations together. Statistical significance is given, where *NS* indicates 'not significant' to p < 0.05. Where statistical significance was approached but not reached, the calculated p value is given *(NS (p value))*.

	**Kendall's tau of Correlation Between Cell Migration Velocity and Receipt of HDF Fluorescence by SAOS-2**	
	Fa	Fmc
**Experiment a**		
Kendall's tau of all Generations Considered Together	0.10	0.13
Statistical Significance of the Above	*NS (0.051)*	*0.009*
Kendall's tau of Starting and First Generations Considered Together	0.10	0.18
Statistical Significance of the Above	*NS (0.088)*	*0.0019*
**Experiment b**		
Kendall's tau of all Generations Considered Together	0.03	-0.004
Statistical Significance of the Above	*NS*	*NS*
Kendall's tau of Starting and First Generations Considered Together	0.02	-0.02
Statistical Significance of the Above	*NS*	*NS*
**Experiment c**		
Kendall's tau of all Generations Considered Together	0.24	0.19
Statistical Significance of the Above	*< 0.0001*	*<0.0002*
Kendall's tau of Starting and First Generations Considered Together	0.25	0.19
Statistical Significance of the Above	*< 0.0001*	*< 0.0002*
**Experiment d**		
Kendall's tau of all Generations Considered Together	0.03	-0.03
Statistical Significance of the Above	*NS*	*NS*
Kendall's tau of Starting and First Generations Considered Together	0.06	0.04
Statistical Significance of the Above	*NS*	*NS*
Kendall's tau of Starting, First and Second Generations Considered Together	0.08	0.01
Statistical Significance of the Above	*0.2*	*NS*
**Experiment e**		
Kendall's tau of all Generations Considered Together	0.08	0.06
Statistical Significance of the Above	*0.045*	*0.11*
Kendall's tau of Starting and First Generations Considered Together	0.003	-0.045
Statistical Significance of the Above	*NS*	*NS*
Kendall's tau of Starting, First and Second Generations Considered Together	0.07	0.04
Statistical Significance of the Above	*NS*	*NS*
**Experiment f**		
Kendall's tau of all Generations Considered Together	0.19	0.15
Statistical Significance of the Above	*0.0017*	*0.013*
Kendall's tau of Starting and First Generations Considered Together	0.17	0.14
Statistical Significance of the Above	*0.0065*	*0.025*
**Experiment g**		
Kendall's tau of all Generations Considered Together	0.1	0.011
Statistical Significance of the Above	*0.0097*	*NS*
Kendall's tau of Starting and First Generations Considered Together	0.05	-0.04
Statistical Significance of the Above	*NS*	*NS*
Kendall's tau of Starting, First and Second Generations Considered Together	0.11	0.02
Statistical Significance of the Above	*0.0069*	*NS*

Very weak correlation of cell migration with HDFs fluorescence was seen in most experiments, but this reached statistical significance in only 5 of 7 of these (a, c, e, f and g). The correlation was strongest for Fa in all experiments other than in 'a' where the strongest correlation was for Fmc. Dependent on which grouping of generations were considered, extremely weak negative correlations were seen for Fmc in four experiments (b, d, e, f), and none of these were statistically significant, so it seems reasonable to reject them.

There was no statistically significant difference between Fa and Fmc. Comparison of Fa with Fmc provided negligible evidence for any persistence of the effect beyond cell division, with a calculated index of 0.14. Overall, data suggest a modest association between fluorescence uptake and cell migration, that is not persistent past cell division.

Table S11. Median values for cell migration velocity in control SAOS-2 and HDFs cultured in isolation, as well as in co-cultures across all experiments. Statistical significance is shown as per Mann Whitney U Test of comparisons between co-cultured cells and control cells cultured in isolation. Where statistical significance was not reached, '*NS*' is shown for 'not significant'.

		Median Cell Migration Velocity (μm/day)	
		SAOS-2	HDFs
Experiment a	Isolated Control Culture	94	456
	Co-Culture	76	329
	Statistical Significance	*0.0003*	*< 0.0001*
Experiment b	Isolated Control Culture	84	287
	Co-Culture	90	304
	Statistical Significance	*NS*	*NS*
Experiment c	Isolated Control Culture	58	244
	Co-Culture	63	172
	Statistical Significance	*0.0179*	*< 0.0001*
Experiment d	Isolated Control Culture	86	327
	Co-Culture	94	217
	Statistical Significance	*NS*	*< 0.0001*
Experiment e	Isolated Control Culture	81	401
	Co-Culture	89	304
	Statistical Significance	*NS*	*<0.0001*
Experiment f	Isolated Control Culture	67	230
	Co-Culture	72	266
	Statistical Significance	*0.0326*	*NS*
Experiment g	Isolated Control Culture	92	316
	Co-Culture	104	333
	Statistical Significance	*0.0060*	*NS*
Medians of Experiments	Isolated Control Culture	84	316
	Co-Culture	89	304

SAOS-2 migration velocity was greater in co-cultures compared with control cultures in 6 of 7 experiments (experiments b, c, d, e, f, g), but this only reached statistical significance in 3 experiments ($p < 0.035$, Mann Whitney U Test), and was not statistically significant when all experiments were considered together ($p > 0.05$, Wilcoxon Signed Rank Test). Despite control and HDFs often differing in a statistically significant manner with regard to cell migration velocity within experiments, there seemed no preference for either control or co-cultured HDFs to have greater migration velocity relative to the other.

Table S12. Kendall's tau for correlation of differences between paired sister cells for cell migration velocity and absolute fluorescence (Fa). Statistical significance *(Stat. Sig.)* is indicated to $p < 0.05$, and *'NS'* for 'not significant' is recorded where statistical significance was not reached.

	First Generation Cells		Second Generation Cells		First and Second Generations of Cells Considered Together	
	Kendall's tau	*Stat. Sig.*	Kendall's tau	*Stat. Sig.*	Kendall's tau	*Stat. Sig.*
Experiment a	-0.11	*NS*	-0.02	*NS*	-0.11	*NS*
Experiment b	-0.05	*NS*	-0.02	*NS*	-0.06	*NS*
Experiment c	0.15	*NS*				
Experiment d	-0.12	*NS*	0.23	*NS*	-0.04	*NS*
Experiment e	0.01	*NS*	0.16	*NS*	0.10	*NS*
Experiment f	0.14	*NS*	0.60	*NS*	0.16	*NS*
Experiment g	0.09	*NS*	0.10	*NS*	0.12	*NS*

No convincing statistically significant correlations were seen.
